# Assessing and Predicting the Water Resources Vulnerability under Various Climate-Change Scenarios: A Case Study of Huang-Huai-Hai River Basin, China

**DOI:** 10.3390/e22030333

**Published:** 2020-03-14

**Authors:** Yan Chen, Yazhong Feng, Fan Zhang, Fan Yang, Lei Wang

**Affiliations:** 1 College of Economics and Management, Nanjing Forestry University, Nanjing 210037, China; fengyazhong@njfu.edu.cn; 2 School of Renewable Natural Resources, Louisiana State University Agricultural Center, Baton Rouge, LA 70803, USA; fzhan14@lsu.edu; 3 Faculty of Business and Economics, Monash University, Melbourne, VIC 3800, Australia; fyan0027@student.monash.edu; 4 Teachers and Teaching Development Center, Nanjing University of Information Science and Technology, Nanjing 210044, China; 001530@nuist.edu.cn

**Keywords:** Huang-Huai-Hai river basin, water resources vulnerability, assessment, scenario prediction, random forest model

## Abstract

The Huang-Huai-Hai River Basin plays an important strategic role in China’s economic development, but severe water resources problems restrict the development of the three basins. Most of the existing research is focused on the trends of single hydrological and meteorological indicators. However, there is a lack of research on the cause analysis and scenario prediction of water resources vulnerability (WRV) in the three basins, which is the very important foundation for the management of water resources. First of all, based on the analysis of the causes of water resources vulnerability, this article set up the evaluation index system of water resource vulnerability from three aspects: water quantity, water quality and disaster. Then, we use the Improved Blind Deletion Rough Set (IBDRS) method to reduce the dimension of the index system, and we reduce the original 24 indexes to 12 evaluation indexes. Third, by comparing the accuracy of random forest (RF) and artificial neural network (ANN) models, we use the RF model with high fitting accuracy as the evaluation and prediction model. Finally, we use 12 evaluation indexes and an RF model to analyze the trend and causes of water resources vulnerability in three basins during 2000–2015, and further predict the scenarios in 2020 and 2030. The results show that the vulnerability level of water resources in the three basins has been improved during 2000–2015, and the three river basins should follow the development of scenario 1 to ensure the safety of water resources. The research proved that the combination of IBDRS and an RF model is a very effective method to evaluate and forecast the vulnerability of water resources in the Huang-Huai-Hai River Basin.

## 1. Introduction

Water resource is widely regarded as among the most important elements to sustain both ecosystems and the economy [1]. The comprehensive management of water resources based on the basin as a unit conforms to the law of natural migration and economic and social characteristics of water resources, which ensures that all the functions of the water resources are addressed. However, due to flood disaster, water shortage, and water pollution, many Chinese river basins are showing varying degrees of water resource system vulnerability. Therefore, it is of great importance for Chinese major river basins to evaluate the water resource vulnerability and reveal the causes [2]. Moreover, climate change also might be a crucial point to affect the water resource system vulnerability. The fifth assessment report of the Intergovernmental Panel on Climate Change pointed out that climate change, population growth, and economic activities all aggravate the adverse impact on water resources systems [3]. Thus, forecasting future water resources vulnerability under the dual influence of human activities and climate change would help policymakers formulate the water resources management strategy in response to future changes in both humanity and climate dimensions. The Huang-Huai-Hai Basin plays a significant strategic role in Chinese economic development [4]. The Gross Domestic Product (GDP), population, and cultivated land area of this region respectively account for 31%, 35%, and 38% of the whole country. However, only 7.2% of the total Chinese water resources are in this region [5]. Obviously, this huge imbalance may cause serious water shortage and water pollution problems. In addition to the results of current human activities, climate change may further aggravate the water resources problems that this area is facing right now and make the whole water resources system even more vulnerable in the future. Based on these facts, there are two necessary things, which are identify the causes of the water resources vulnerability and anticipate water resources vulnerability in different scenarios in the future in Huang-Huai-Hai basins. 

The research on water resources vulnerability starts from groundwater resources and gradually develops to surface water resources and the overall system. In the early 1970s, the concept of groundwater vulnerability was proposed [6]. Since the 1990s, some scholars began to research the surface water resources vulnerability and water resources system [7,8]. In recent years, there are some new changes in the research object. The original study object of water vulnerability was inland water, which has since grown to include marine and mountain water. The research of inland water mainly focused on the specific river basin and area, which includes the spatiotemporal analysis, the water resources vulnerability assessment, and classification. Research of assessment and classification could help provide some reference for the formulation of water resources management policies [9]. Marine water research focuses on cross-border water resources management and protection. Some scholars have empirically analyzed the current and future vulnerability of water resources in the Adriatic, where the point of penetration is the impact of climate change on water availability and water security [10,11]. Other scholars believe that mountains are important water towers, and put forward the global water tower index, which is used to assess the vulnerability and importance of water resources [12]. The empirical research area covers many areas from Australia in the southern hemisphere to the Arctic [13,14]. Research methods include qualitative analysis and quantitative analysis. The qualitative analysis is mainly to explore the influencing factors that lead to the vulnerability of water resources, which is focused on two aspects: natural factors and social factors [15]. Some qualitative analysis is to develop a framework for assessing water vulnerability [16], and quantitative analysis mainly includes two kinds of research methods: the function method and index method [17,18]. The function method mainly studies the mechanism of water resource vulnerability. The evaluation index method mostly adopts TOPSIS (Technique for Order Preference by Similarity to an Ideal Solution) [19] and the entropy weight method [20]. 

In 1996, the Intergovernmental Panel on Climate Change (IPCC, Geneva, Switzerland) pointed out that the research on WRV should consider the climate change influence [21]. After that, the vulnerability of water resources under the influence of climate change has become a hot issue in academic research. There are also some scholars who research on the regional and global water resources vulnerability under the both impact of human social–economic activities and climate changes [22,23]. In addition, the future research trend is to study the security of water resources and other energy sources in coordination [24]. It includes not only studying the vulnerability of the water resources system, but also links water resources with energy and food [25,26]. The Huang-Huai-Hai basins have a special geographical location and severe water resources research, which has attracted extensive attention in the field of academic research. Some scholars have studied water resources vulnerability at one of the three basins [27,28]. At present, most of the research studies focus on the changes of hydrological and meteorological indicators over the past 50 years in the Huang-Huai-Hai River Basin, such as the water yield coefficient, the spatial and temporal changes under extreme precipitation, the combined characteristics of drought on multiple time scales, and the trends in extreme temperature indices [29,30,31,32]. In addition, some studies have analyzed the climate change influence and runoff in three basins’ water resources [33,34].

After decades of research assessing the water resources vulnerability, the scope of the research object is gradually expanding from the initial groundwater resources to the surface water, regional, and river basin water resources. Specifically, the evaluation index system is the mainstream method to evaluate the vulnerability of water resources, in which the coverage of the evaluation index system has gradually changed from the original simple water quality index to a combination with water quality, water quantity, disaster, and other indicators. Nowadays, more and more scholars are paying attention to the dynamic changes of water resources vulnerability (WRV), which included the influence of human socio-economic activities and climate change. The Huang-Huai-Hai River Basin remains a hot research spot in the field of water resources research in China. It is because of its strategic position in Chinese social–economic development and severe water resources problems. Most existing studies of the influence of climate change on the water resources in this area focused on the trends of single hydrological and meteorological indicators. No existing research ever comprehensively identified the causes of water resources vulnerability in each of these three river basins, while assessing and predicting the impacts of human social–economic activities and climate change. 

The main purpose of this paper is to assess and forecast the water resource vulnerability in the Huang-Huai-Hai River Basin under human socio-economic activities and various climate change schemes. The context of paper is organized as follows. The first part is the introduction, which mainly describes the existing problems of water resources in the Huang-Huai-Hai River Basin, and the research progress of water resources vulnerability. Section 2 includes the data and methodology, which mainly introduces the general situation of the research area, data sources, and the methods used in this paper. In the Methodology section, we mainly describe the combination of Improved Blind Deletion Rough Set (IBDRS) and the random forest (RF) model. Section 3 features the results and discussion; we use the IBDRS to reduce the dimension of the index system and the RF model to evaluate and predict the vulnerability of water resources in the Huang-Huai-Hai basin. Section 4 is the conclusion, which mainly summarizes the research process and expounds the contribution of this paper.

## 2. Data and Methodology

### 2.1. Research Area

**Figure 1:**
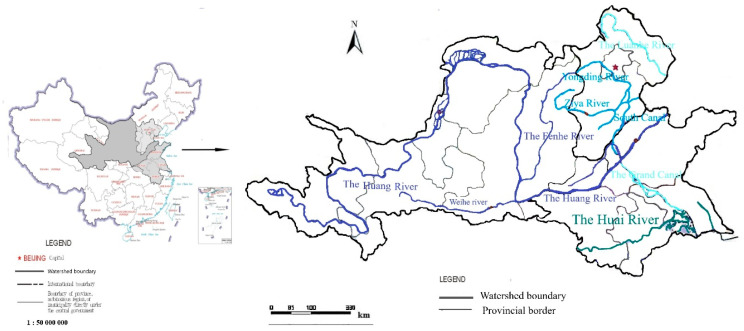
Distribution map of the Huang-Huai-Hai River Basin.

As shown in Figure 1, the Huang-Huai-Hai River Basin consists of three main basins, namely, the Huang River Basin, the Huai River Basin, and the Hai River Basin. These three river basins are classified as the first-grade water resource areas in China. The whole region covers 14 provinces and municipalities, i.e., Qinghai, Gansu, Ningxia, Sichuan, Shaanxi, Shanxi, Inner Mongolia, Hebei, Henan, Shandong, Anhui, Jiangsu, Tianjin, and Beijing. The total area of the region is 1,445,000 km^2^, accounting for approximately 15% of China’s total land area. According to the topography, this region can be divided into four regions from east to west: The Huang-Huai-Hai Plain, the Loess Plateau, the Inner Mongolia Plateau, and the Qinghai-Tibet Plateau. The elevation of these four areas decreases along the way from west to east as all these three rivers flow toward the east in general. Tremendous variations in latitude, longitude, and altitude result in huge differences in terms of climate over the whole region. According to the annual precipitation, this region covers four different climate zones: humid, semi-humid, semi-arid, and arid zone. The annual average precipitation is 556.0 mm and the evaporation is 1699.5 mm. The Huang-Huai-Hai Basin is a major grain production area and energy base.

The Huang-Huai-Hai Basin is facing severe water resources problems primarily due to a huge imbalance between the limited gross water amount and prosperous social economic activities in this region. The water resources per capita are 462 m^3^ in the Huang-Huai-Hai basin, which is only 21% of the national average level. Furthermore, the insufficient amount of water resources is not the only problem. In fact, this region is also facing a serious problem of unevenly distributed precipitation, which means that this region may suffer from both flooding and drought at the same time. According to statistics from other studies, there are two droughts every three years in this region. Due to the joint action of human activities and climate change, the underlying conditions of these basins have changed significantly, resulting in profound changes in the relationship between precipitation and water resources, so the water resources have been greatly reduced. Hence, as the water resources situation become more vulnerable than in the past, the water resources management in this region is facing great challenges.

### 2.2. Data Sources

For the empirical part of this study, our research period is from 2000 to 2015. For the scenario predictions part, we primarily focus on the water vulnerability at the time points of the years 2020 and 2030, respectively. The data for these two parts of studies are obtained via different ways.

We choose 2000–2015 as the research period for two important reasons: one is to consider the availability of data, the other is the effect of water resources policy of the State Council. The State Council issued the “State Council’s Views on the Strictest Water Resources Management System (SWRMS)” in document [35]. We chose the research period that can cover this event, which enables us to examine the effect of this new policy. Data are collected from each of these three basins. Since there are 16 observations for each basin, we have a total of 48 observations for all three river basins combined. For each observation, we have considered 24 individual indicators as observed variables. The data sources included the following: “The Water Resources Bulletin of the Huang-Huai-Hai Basin” (2000 to 2015), “The China Environmental Statistics Yearbook” (2000 to 2015), and “The Chinese Statistical Yearbook” (2000 to 2015). 

Since the climate change scenarios are not certain in the future, we mainly set up three future scenarios. For each scenario, the value of all 24 variables will be changed following certain rules. The indicators of the three scenarios in the future mainly include two categories; one is the indicators that are greatly affected by climate, and the other is the indicators that are greatly affected by human social and economic activities. For these two types of indicators, we adopt different scenario data settings.

The first type of scenario data, which are greatly affected by climate, are mainly collected from the research results of Xia Jun’s team and others [36,37,38]. Specifically, according to the Fifth Assessment Report of the IPCC, the precipitation and runoff data of the Huang-Huai-Hai Basin in 2020 and 2030 under different climate change scenarios were collected. Scenarios 1, 2, and 3 in this paper correspond to the three concentration emission scenarios of Representative Concentration Path (RCP) 2.6, RCP 4.5, and RCP 8.5 in the report, respectively. For example, the calculation of water production modulus (*A_1_*) is based on the precipitation and water production coefficient data under three concentration discharge scenarios to calculate the total water resources. The *A_1_* is obtained by dividing the total water resources of the basin by the area of the basin.

The second type of scenario data is greatly influenced by human social and economic activities. The forecast data of these indicators mainly come from the water resource management objectives in 2020 and 2030 of the “State Council’s Views on the SWRMS” and the comprehensive planning of the three river basins (2012–2030). Among them, the index data of economic activity impact in scenario 1 can fully achieve the target data set in the SWRMS, while scenario 2 and scenario 3 are determined according to the proportion of the data in scenario 1. For example, the qualification rate of water quality in the water function area (*B_3_*) of each basin under scenario 1 can fully achieve the planning objectives, while scenario 2 and scenario 3 are calculated according to the current progress and 80% or 60% of the planning objectives. According to the above scenario setting method, we set the index data of three scenarios in the future in 2020 and 2030. The scenario prediction data and prediction process are shown in Table A1 in the Appendix A.

### 2.3. Methodology

**Figure 2:**
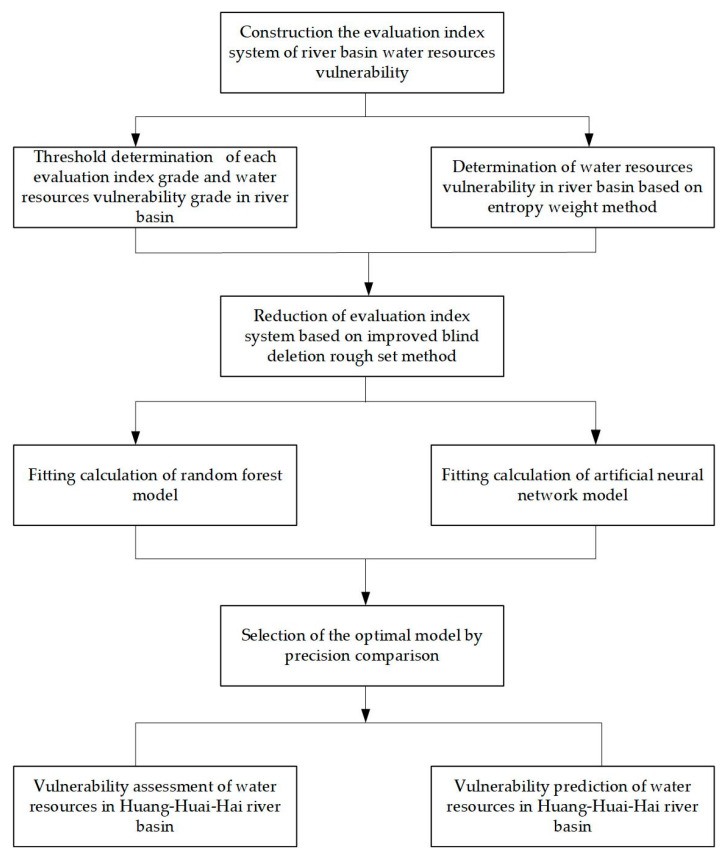
Flow chart of vulnerability assessment and prediction method of water resources in the three river basins.

The research method and flow chart of this paper are shown in Figure 2, which has three important steps. Firstly, we construct the evaluation index system. Secondly, using the Improved Blind Deletion Rough Set (IBDRS) method to reduce dimension and simplify the original index system. Afterward, the RF model and artificial neural network (ANN) model are used to train the original data of the reduced indicators and the results. By comparing the fitting accuracy of the two models, we choose the one with the higher fitting accuracy as the model used for evaluation and prediction.

#### 2.3.1. Evaluation Index System

In this paper, the index system method is used to evaluate and forecast the river basin water resources vulnerability. On the basis of the causes and manifestations of the water resources vulnerability, we divide the water resource vulnerability index (WVI) into three secondary indexes, which are water shortage vulnerability (WSVI), water pollution vulnerability (WPVI), and water-related natural disaster vulnerability (WDVI) [39]. Then, we divide each secondary indicator into four three level indexes, namely, the pressure, state, impact, and response. Finally, we choose 24 indicators as the initial evaluation index set. The evaluation index system is shown in Table 1.

#### 2.3.2. Index Reduction Method

In our original comprehensive evaluation index system, the number of evaluation indexes is huge, which will affect the efficiency of evaluation and prediction. Therefore, we need to reduce the dimension of the original index system. The main principles in the process of reducing indicator dimensions are to maintain the same accuracy as the original index and ultimately improve the efficiency of evaluation and prediction. To balance the interpretability and predicting performance and to keep the RF and ANN models comparable, we must make sure that all models we wanted to compare have the same input variables. Therefore, we disabled the variable selection process embedded in the RF and ANN models but use an independent variable selection process. 

Specifically, in this study, the IBDRS is used to screen the evaluation indicators. Rough set is a mathematical tool for dealing with incompleteness and uncertainty. This method is widely used in many fields of natural science, engineering technology, and social science [40]. Compared with the common methods of index dimension reduction such as traditional rough set (RS), analytic hierarchy process (AHP), and principal component analysis (PCA), the IBDRS method achieves a perfect balance in the ability of data mining, variable interpretation, and subjective will.

For example, in the process of reduction, the RS finds the hidden knowledge and rules through the analysis of each index data, which is scientific. However, at the same time, important indicators may be deleted. In addition, the AHP method is very subjective in the process of index deletion, which depends on the judgment of experts. However, the PCA method is relatively objective, but in the process of dimensionality reduction, there are new composite indicators, so it is impossible to analyze the original indicators. For the reason of retaining some necessary attributes in the system subjectively while reducing the data dimension effectively, we choose the IBDRS method for this study. 

The main ideas of IBDRS are as follows. Firstly, according to the classification standard of each evaluation index, the original numerical data and the evaluation results calculated by the entropy weight method are discretized to form a decision table. Then, we use the traditional RS method to reduce the dimension of the index and find the core of the index set. Thirdly, we add important indicators to the core. At the same time, the balance of the number of indicators between the secondary indicators should be considered. After adding an indicator, the equivalence relationship between the subset before adding and the subset after adding will be verified according to the concept of rough set. The detailed principles and procedures of this method can refer to the work by Pawlak [40], and the detailed calculation steps of this method are shown in reference [39]. 

#### 2.3.3. Random Forest and Artificial Neural Network Models

At present, there are two main types of prediction methods. The first kind of forecasting method is the traditional statistical method, mainly including a regression analysis model, time series analysis model, and other methods. The advantage of these methods is that the structure is simple and easy to identify, but it is difficult to achieve ideal results in the data is in non-linear form [41]. The second is machine learning models; some of commonly used methods fall into this category include ANN, RF, and Support Vector Machine (SVM) models [42]. At present, the application of machine learning models in the field of evaluation and prediction is booming. 

RF is a combinatorial algorithm based on multiple categorical and regression trees (CART) first proposed by Breiman in 2001 [43]. RF has been widely used in classification, evaluation, and prediction. In recent years, many studies have also been applied in the fields of hydrology and water resources. In the classification, a novel hierarchical object-based Random Forest classification approach can be used to distinguish different land cover types, which have accuracy rates over 90% [44]. RF could also establish a basin hydrological evaluation model by determining the weight of the index, and the accuracy rate is higher than the entropy weight method [45]. In addition, RF in hydrological data prediction has a strong advantage, and its prediction results are better than Poisson regression [46], which could also effectively divide flood-prone areas [47,48]. However, the research trend of RF is more combined with other model methods, and the mixed model can make up for the shortcomings of a single model. In the mixed model, some scholars have tried to combine RF with the Wavelet model, Kernel Ridge Regression (KRR), and other models [49,50]. Through empirical analysis, it is confirmed that these composite models are better than a single model.

The random forest algorithm is based on statistical theory, using the bootstrap resampling method to extract multiple samples from original samples. For each extracted sample, a decision tree is constructed to generate multiple complete depth tree models. When the random forest model is used for predicting purpose, the final predicted value is obtained by averaging the predicted value of multiple tree models. On the other hand, when the random forest method is used to solve classification problems, the final prediction can be generated by majority voting. Random Forest is a combination forecasting model, which can be regarded as a strong predictor integrated by many weak predictors (decision trees). These weak predictors complement each other and can reduce the impact of single predictor errors, thus improving the accuracy and stability of prediction. The random forest approach is robust to both outliers and noise multiple collinearities problems [51]. Therefore, random forest has a good performance in multivariate prediction and its interpretation, so that it has been widely used in many fields such as medicine, biology, and so on [52]. Actually, RF is almost similar to a black box. It cannot control the internal operation of the model. If there are too many training samples, there might be many similar decision trees during the training process, which mask the true results. Besides, for small or low-dimensional data, overfitting may also occur on some noisy classification or regression problems. However, the accuracy of the model can be improved by adjusting the parameters *ntree* to avoid over fitting. RF model has a strong advantage in dealing with large-scale multivariate data, which meets the requirements of water resources vulnerability assessment and prediction involving multiple indicators and complex data processing.

ANN is a non-linear and adaptive information processing system based on the research results of modern neuroscience. ANN has low requirements for input information, which has many applications in the field of hydrological prediction. It could effectively detect flood-prone areas and be used as a decision support system for the comprehensive evaluation and management of water resources [53]. Since the learning rate of the artificial neural network is fixed, the convergence rate of the network is slow and needs a long training time. So, in recent years, some scholars try to use some algorithms combined with the ANN to speed up its convergence rate, such as AEEMD–ANN (an adaptive ensemble empirical mode decomposition with the ANN), SSA–ANN (a singular spectrum analysis with the ANN), PSO–ANN (Particle Swarm Optimization PSO with the ANN), and other optimization models are proposed [54,55,56].

The artificial neural network model emerged from the field of artificial intelligence in the 1980s [57]. It processes information by simulating the structure and function of a human brain neural network. Artificial neural network is a non-linear system consisting of many interconnected neurons. It is not implemented step by step according to a given procedure, but is trained, studies, and then repeatedly modifies the weights of each neuron. It is because the learning rate of ANN is fixed and the convergence speed of the network is slow, which requires a long training time. However, it could be improved by changing the learning rate or the adaptive learning rate. In addition, due to the lack of training samples or external noise and other factors, the neural network will appear to have an “over fitting” phenomenon in the training process. At present, the artificial neural network model is widely used in many fields such as economy, biology, medicine, and so on. It realizes many functions such as recognition, evaluation, prediction, classification, etc. 

We intend to use the RF and ANN to carry out simulation training between the original data and the vulnerability. By comparing the fitting ability of the two models, we find out the best model with the highest fitting accuracy as the evaluation and prediction model.

## 3. Result and Discussion

First of all, the IBDRS method is used to reduce the dimension of the initial index system, and the index system after dimension reduction has the same evaluation ability as the initial index. Then, we compare the fitting accuracy of the RF and ANN-based reduced set of variables. Finally, the model with the higher fitting accuracy is selected from these two models to forecast the vulnerability of three river basins in 2020 and 2030 under various scenarios.

### 3.1. Dimension Reduction of Evaluation Index

Our preliminary analysis has shown that some variables are highly correlated with others, which enables us to use the dimension reduction approach aiming at saving computational power while maintaining prediction performance. In the process of reducing the original evaluation index set by the IBDRS method, it is necessary to discretize the original data and obtain a decision table C. The decision table C contains two kinds of data: conditional attribute data and decision attribute data. The first category is the condition attributes, which are obtained by the discretization of the original data of each index using the k-means clustering algorithm. The continuous data are transformed into integers between 1 and 4, and the conditional attribute values in the decision table are obtained. The biggest advantage of K-means is that it is easy to understand, simple, and fast to run [58]. We use SPSS 21 software to carry out the k-means clustering process and specify the value of K as 4. However, the other category is the decision attribute, which is determined by the entropy weight method and pre-determined index threshold. We use the entropy weight method and threshold value of 24 original evaluation indexes to determine the decision attributes. The specific steps are listed as follows. Firstly, the weights of the dimensionless indexes are calculated by using the entropy weight method, and seven evaluation grade threshold tables are calculated according to the thresholds of 24 evaluation indexes, as shown in Table 2. Then, according to the weights and dimensionless values of 24 indicators in the three basins from 2000 to 2015, the comprehensive evaluation values of water resources vulnerability in the three basins are obtained by weighting calculation. The decision attributes of the three basins are obtained by judging the level of the comprehensive evaluation value according to the threshold levels in Table 2.

The implementation process of reducing the dimension of evaluation index by the IBDRS method is presented as follows:

We define the original conditional attribute set (original evaluation index system) as *C* and the initial reduction index set as *B*. Select five indexes from WSVI, WPVI, and WDVI respectively to establish the initial reduction index set B, and $\;B=\left\{A_{1},A_{3},A_{5},A_{6},A_{7},B_{2},B_{3},B_{4},B_{5},B_{8},C_{1},C_{3},C_{4},C_{6},C_{8}\right\}$

Verify the validity of equation $pos_{B}\left(D\right)=pos_{C}\left(D\right)$. If the above equation holds, it shows that index set B has the same classification ability as initial condition attribute set *C*, so there is no need to add another indicator to *B*.

We use the IBDRS method to verify the necessity of each evaluation index in *B*. In the process of deleting indicators, in order to keep the balance of the number of reduced indicators in WSVI, WPVI, and WDVI as far as possible, the indicators are deleted one by one from WSVI, WPVI, and WDVI or cyclic verification. The main steps are listed as follows.

**Step 1**: Remove the indicator $A_{5}$, and then the equation $pos_{B-A_{5}}\left(D\right)=pos_{B}\left(D\right)$ holds up, indicating that the indicator $A_{5}$ can be removed. In this way, the initial index set *B* can be reduced to $B=\left\{A_{1},A_{3},A_{6},A_{7},B_{2},B_{3},B_{4},B_{5},B_{8},C_{1},C_{3},C_{4},C_{6},C_{8}\right\}$.

**Step 2**: Remove the indicator $B_{4}$, and then the equation $pos_{B-B_{4}}\left(D\right)=pos_{B}\left(D\right)$ holds up, indicating that the indicator $B_{4}$ can be removed. In this way, the initial index set *B* can be reduced to $B=\left\{A_{1},A_{3},A_{6},A_{7},B_{2},B_{3},B_{5},B_{8},C_{1},C_{3},C_{4},C_{6},C_{8}\right\}$.

**Step 3**: Remove the indicator $C_{4}$, and then the equation $pos_{B-C_{4}}\left(D\right)=pos_{B}\left(D\right)$ holds up, indicating that the indicator $C_{4}$ can be removed. In this way, the initial index set *B* can be reduced to $B=\left\{A_{1},A_{3},A_{6},A_{7},B_{2},B_{3},B_{5},B_{8},C_{1},C_{3},,C_{6},C_{8}\right\}$.

**Step 4**: Remove the indicator $C_{6}$, and then the equation $pos_{B-C_{6}}\left(D\right)=pos_{B}\left(D\right)$ holds up, indicating that the indicator $C_{6}\;$ can be removed. In this way, the initial index set *B* can be reduced to $\;B=\left\{A_{6},A_{7},B_{3},B_{5},C_{3}\right\}$, and $\left|pos_{B}\left(D\right)\right|=48$.

$$pos_{\left(B-A_{6}\right)}\left(D\right)\neq pos_{B}\left(D\right),\;\left|pos_{\left(B-A_{6}\right)}\left(D\right)\right|=45$$

$$pos_{\left(B-A_{7}\right)}\left(D\right)\neq pos_{B}\left(D\right),\;\left|pos_{\left(B-A_{7}\right)}\left(D\right)\right|=43$$

$$pos_{\left(B-B_{3}\right)}\left(D\right)\neq pos_{B}\left(D\right),\;\left|pos_{\left(B-B_{3}\right)}\left(D\right)\right|=45$$

$$pos_{\left(B-B_{5}\right)}\left(D\right)\neq pos_{B}\left(D\right),\;\left|pos_{\left(B-B_{5}\right)}\left(D\right)\right|=39$$

$$pos_{\left(B-C_{3}\right)}\left(D\right)\neq pos_{B}\left(D\right),\;\left|pos_{\left(B-C_{3}\right)}\left(D\right)\right|=44$$

At this time, B is the smallest reduction set extracted from the original decision table. We define B as a core, and every indicator in this core is necessary. $\mathrm{And}\;B=\left\{A_{6},A_{7},B_{3},B_{5},C_{3}\right\}$.

We find out that there is an unbalanced distribution of the number of indicators in the index set B. This situation may affect the prediction accuracy of WSVI, WPVI, and WDVI. Therefore, based on the experience judgement of experts, we add 7 indicators such as $\left\{A_{1},A_{8},B_{2},B_{4},C_{6},C_{7},C_{8}\right\}\;$ to the simplest core indicator set $\left\{A_{6},A_{7},B_{3},B_{5},C_{3}\right\}$ and form the final prediction indicator set B, $B=\left\{A_{1},A_{6},A_{7},A_{8},B_{2},B_{3},B_{4},B_{5},C_{3},C_{6},C_{7},C_{8}\right\}$.

### 3.2. Selection of Evaluation and Prediction Models

In this part, we use the RF and ANN models to fit and choose the model with better fitting and prediction ability as the prediction model.

#### 3.2.1. Optimization of Model Parameters

**Figure 3:**
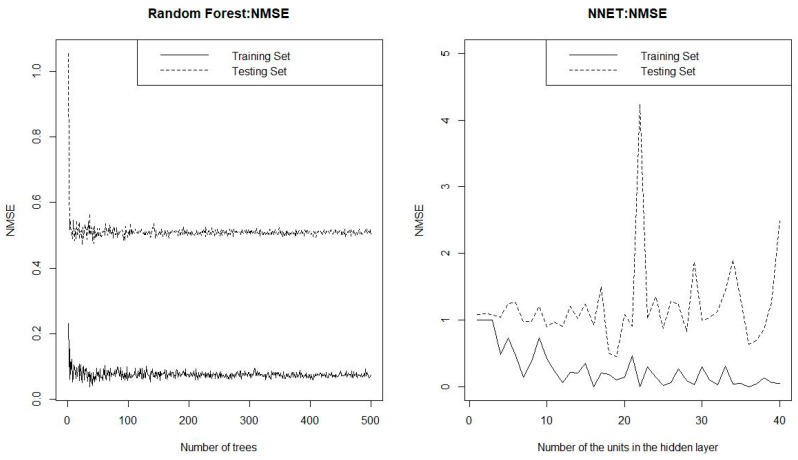
Parameter setting process chart of the random forest (RF) model (**a**) and artificial neural network (ANN) model (**b**).

This part mainly we use the reduced index set of 12 indicators, which includes variables *A_1_*, *A_6_*, *A_7_*, *A_8_*, *B_2_*, *B_3_*, *B_4_*, *B_5_*, *C_3_*, *C_6_*, *C_7_*, and *C_8._* The *ntree* and *mtry* are the main parameters that need to be set in the RF model. The parameter *ntree* represents the number of decision trees, and *ntree* > 100. The parameter *mtry* is the most sensitive parameter in the RF model, which represents the number of variables selected when the nodes of the decision tree split. Throughout the generation process of a random forest, *mtry* remains unchanged. When the number of original variable sets is *n*, it is recommended that *mtry* be n/3. We use the bootstrapping method to determine the optimal parameters by comparing the OOB (out of bag) errors under different parameters. In another word, the optimal values of *ntree* and *mtry* are determined when they lead to the smallest OOB error. As shown in Figure 3, on the left, the prediction accuracy of RF becomes higher and more stable as the number of trees increases. When *ntree* takes 500 and *mtry* takes 4, the OOB error is the smallest. The parameters for an ANN model are mainly the number of hidden layers. As one can see from Figure 3 on the right, when the number of hidden layer increases, its accuracy will not raise substantially. When the number of hidden layers of the neural network is 9, the accuracy is the best.

#### 3.2.2. Evaluation of Fitting Accuracy of Models

This paper evaluates the goodness of fit of the aforementioned two models based on following two indicators: Mean Square Error (*MSE*) and Normalized Mean Square Error (*NMSE*). We use the method of 10-fold cross-validation to calculate the mean values of *MSE* and *NMSE* as the accuracy criteria. The formulas for these two fitting accuracy evaluation indicators are as follows.

(1) $$MSE=\frac{1}{n}\sum {\left(\overset{\wedge }{y_{i}}-y_{i}\right)}^{2}$$

(2) $$NMSE=\frac{\sum {\left(y_{i}-\overset{\wedge }{y_{i}}\right)}^{2}}{\sum {\left(y_{i}-\bar{y_{i}}\right)}^{2}}$$

The values of *MSE* and *NMSE* indicate the differences between the predicted and actual values of the model. The range of the two indicators is usually 0–1, and the smaller the calculated value of the two indicators, the better the performance of the model.

We evaluate the fitting accuracy of the two models by comparing the MSE and NMSE results of the two models. It can be seen from Table 3 that the RF model shows superior performance compared to the ANN model. Therefore, we choose the RF as the assessment and prediction model. 

**Figure 4:**
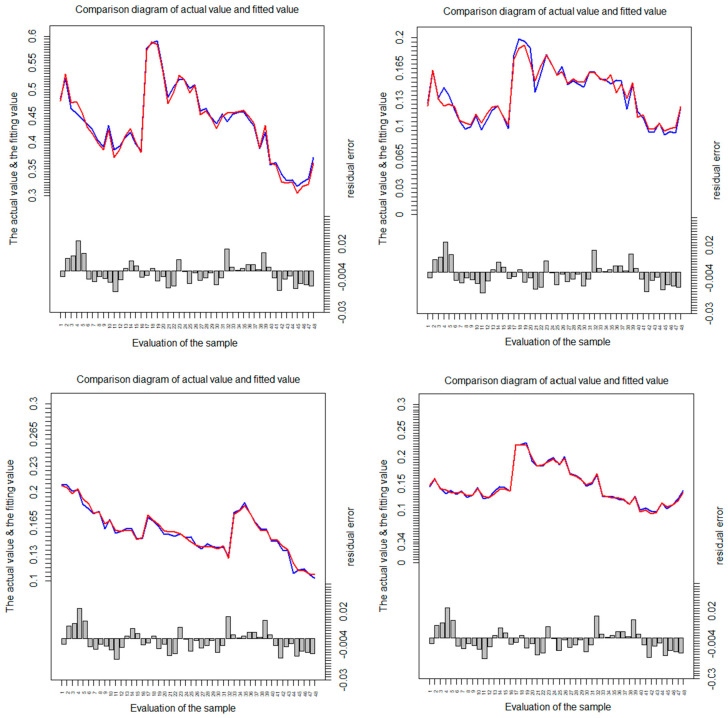
Comparison chart between the fitting value and the actual value in the Huang-Huai-Hai Basin during 2000–2015.

RF were used to calculate the vulnerability in the Huang-Huai-Hai Basin during 2000–2015, including WVI, WSVI, WPVI, and WDVI. We compare the fitted values with the actual value, as shown in Figure 4, where the blue line represents the vulnerability value calculated by the original evaluation index system, and the red line represents the numerical curve fitted by the reduced dimension index and the RF method. As shown in Figure 4, the fitting calculation effect of the RF model is better. 

### 3.3. Assessment of Water Resources Vulnerability in Huang-Huai-Hai Basin

After variable selection, there are 12 indicators that are supplied into a random forest model to evaluate water resources vulnerability in the Huang-Huai-Hai Basin. The results are shown in Table 4.

#### 3.3.1. Trend Analysis of the WVI in Huang-Huai-Hai Basin

**Figure 5:**
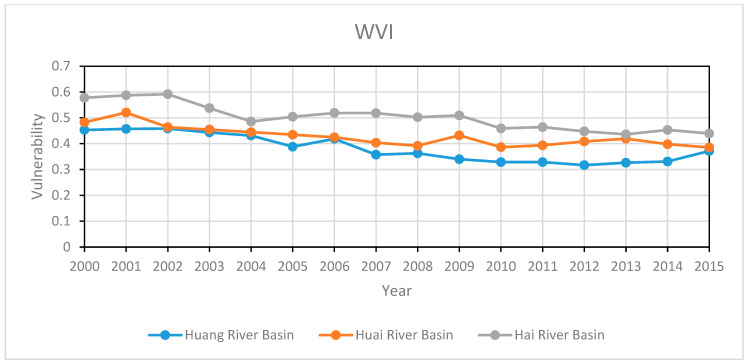
Trend chart of WVI in Huang-Huai-Hai Basin during 2000–2015.

According to the calculation results in Table 4, we have drawn the trend chart of WVI in the Huang-Huai-Hai Basin, as illustrated in Figure 5.

We can see that the values of WVI in the three basins all have been decreased slightly from 2000 to 2015. In general, these results show that the level of WRV in the Huang-Huai-Hai Basin has increased during the 16-year period. 

The WVI in the Huang River Basin was grade 4 during 2000–2006, which is considered a moderate level of vulnerability. During 2007–2014, the vulnerability level was at level 3, falling into the category of moderate to low vulnerability. It can be seen that the WVI has been alleviated in the Huang River Basin during the 16-year period. 

The WVI of the Huai River Basin was grade 4 during 2000–2015, which is considered moderate vulnerability. From the perspective of vulnerability level, there is no significant improvement. However, the absolute value of vulnerability still shows a decreasing trend, indicating small improvements. 

Compared with the other two basins, the WVI in the Hai River Basin is more severe. From 2000 to 2009, the level of WVI in the Hai River Basin was at level 5, which is classified as moderate to high vulnerability. The situation has been improved during the time period between 2010 and 2015, but it is still staying at level 4 (moderate vulnerability).

In general, our study has found that the WVI in the Huang River Basin is the best one among these three river basins. For the other two, Huai River Basin is at a better position than the Hai River Basin in terms of water resource vulnerability.

#### 3.3.2. Cause Identification of Vulnerability in Huang-Huai-Hai Basin

From the calculation results and comparative analysis of WVI, WSVI, WPVI, and WDVI in the three river basins, we found that the WSVI and WDVI are the highest in the Hai River Basin. So, the key vulnerability of the Hai River Basin is caused by water shortage, flood, and drought disaster. In recent years, the water resources vulnerability has been alleviated to some extent in the Hai River Basin. The main reasons are the irrigation of the Huang River diversion and the start-up of the Phase I of South-to-North Water Transfer Project. Since the opening of the South-to-North Water Diversion Project, by the end of 2019, more than 300 billion m^3^ of water had been transferred to Beijing, Tianjin and Hebei, which greatly alleviated the water shortage and drought in the Hai River Basin.

In addition, the water pollution is the key vulnerability factor in the Huai River Basin, which has become an important factor of the basin.

We use 12 evaluation indexes after dimensionality reduction and the RF model to evaluate the water resources vulnerability in the three basins. The error between the evaluation results and the original 24 evaluation indexes is very small. This shows that our reduced index system is more representative and concise. In addition, through the results of key vulnerability analysis, we can find that the index system constructed from the three aspects of water quantity, water quality, and disaster has more advantages than the other functional methods and index systems. This index system is conducive to the cause analysis of the vulnerability of each river basin and the key governance.

Through the trend analysis of the three basins from 2000 to 2015, it can be found that the vulnerability levels of water resources in the Huang River Basin and Hai River Basin have increased by one level after 2007 and 2010 respectively, while the vulnerability levels in the Huai River Basin have not changed and are still at level 4.

The SWRMS issued by the State Council started in 2012, but before and after 2012, the vulnerability of the three basins did not change significantly. However, this paper cannot evaluate the effectiveness of this policy system. The main reason is that the effect of the policy needs to be monitored and observed for a longer period of time. In 2022, we will evaluate the effect of the implementation of the policy for 10 years and study the changes of vulnerability levels before and after the implementation of the policy.

### 3.4. Scenario Prediction of WRV in Huang-Huai-Hai Basin

In the fifth assessment report, the IPCC set four scenarios named the Representative Concentration Path (RCP), namely, Mitigation Emission Path (RCP 2.6), Middle Stability Emission Path (RCP 4.5), High and Stable Emission Path (RCP 6.0), and High Emission Path (RCP 8.5). In these scenarios, the future climate change is predicted solely on the basis of emission changes, while other socio-economic conditions are not considered [33].

On the basis of the three emission paths of RCP 2.6, RCP 6.0, and RCP 8.5, this paper adds the influence of human social and economic activities. We set up three scenarios, namely scenario 1, scenario 2, and scenario 3; the WVI, WSVI, WPVI, and WDVI in the Huang-Huai-Hai Basin in 2020 and 2030 were calculated under three scenarios, as shown in Table 5.

#### 3.4.1. Water Resources Vulnerability Prediction under Scenario 1

Climate change in scenario 1 is based on the RCP 2.6 climate concentration path in the fifth IPCC report, i.e., by 2100, the average temperature rise will be controlled within 2.0 °C, and the radiation forcing will be stable at 2.6 W/m^2^. The changes caused by the impact of human social and economic activities in scenario 1 are the values of indicators under the three red lines of the SWRMS issued by the State Council in 2012.

From Table 5, it can be seen that if each basin develops according to scenario 1, the water resources vulnerability in the three basins will be significantly improved in 2020 and 2030. The WVI, WSVI, WPVI, and WDVI will reach level 3 in the Huang River Basin in 2020. By 2030, the WVI, WPVI, and WDVI can even reach level 2 in the Huang River Basin, which belongs to the mild vulnerability level.

The WVI, WSVI, WPVI, and WDVI can basically reach the level 3 (moderate to mild vulnerability) in the Huai River Basin in 2020 and 2030.

In 2020, the WVI, WSVI, WPVI, and WDVI will at level 4, level 4, level 3, and level 5 respectively in the Hai River Basin. In 2030, the WVI, WSVI, WPVI, and WDVI will at level 3, level 4, level 3, and level 3 respectively in the Hai River Basin.

#### 3.4.2. Water Resources Vulnerability Prediction under Scenario 2

Climate change in scenario 2 is based on the RCP 4.5 climatic concentration path in IPCC’s fifth report, i.e., radiation forcing stabilizes at 4.5 W/m^2^ by 2100. The change of human activities in the scenario 2 is that the indicators of each basin can reach 80–90% of the three red-line control of the SWRMS issued by the State Council in 2012.

Under Scenario 2, the water resources vulnerability did not change significantly in 2020 and 2030 compared with the level of 2015 in the Huang-Huai-Hai Basins. Especially in the Huai River Basin and Hai River Basin, the vulnerability of water resources is basically at levels 4 and 5, which belongs to the moderate to high vulnerability level.

#### 3.4.3. Water Resources Vulnerability Prediction under Scenario 3

Climate change in scenario 3 is based on the RCP 8.5 climate concentration path in the fifth IPCC report, which assumes that there is a lack of policies to deal with climate change, low rate of technological innovation, the largest population, and slow energy improvement, all of which lead to high greenhouse gas emissions and long-term high energy demand; i.e., by 2100 radiation is forcibly increased to 8.5 W/m^2^. The change of human activities in scenario 3 is to develop the economy in an extensive way. Without paying attention to environmental protection, the indicators can only reach the level of 60–80% required by the three red lines.

Under scenario 3, the water resources vulnerability tends to deteriorate in 2020 and 2030 compared with the base year of 2015 in the Huang-Huai-Hai Basin. The vulnerability of water resources in the three basins in 2020 and 2030 is basically at levels 4 and 5, which belongs to moderate to high vulnerability.

In this paper, the reduced-dimensional index and the RF model are used to predict the water resources vulnerability of the three basins in 2020 and 2030. It is very simple and convenient, because we can directly calculate the vulnerability value and level by substituting the predicted index value into the trained RF model without calculating the weight of each index.

However, this method will also be affected by the accuracy of scenario prediction data of each indicator. In this paper, the forecast value of indicators is calculated according to some existing research results and the realization proportion of 2020 and 2030 planning goals of each basin. In the future, we can further study the prediction methods of each index. In the future, detailed research can be carried out on the prediction of each index.

Through the scenario prediction analysis, we can find that if we develop according to scenario 1, the vulnerability level of water resources in the three basins will be improved in 2020 and 2030. Therefore, we should develop in accordance with scenario 1 to ensure the future water resources security of the river basin, and each river basin should achieve the established goals.

## 4. Conclusions

This paper constructs an evaluation index system from the causes of the vulnerability of water resources. Then, the IBDRS method is used to reduce the dimension of the index, and the reduced dimension index has the same evaluation ability as the original index. Furthermore, by comparing the fitting ability of RF and ANN, the RF model was found to be more accurate and thus was used to evaluate and predict the vulnerability of water resources in the Huang-Huai-Hai Basin.

This article draws the following important conclusions. Firstly, the values and levels of WRV in the three river basins decreased during 2000–2015, indicating that the WRV in the three river basins has improved. From the three aspects of water quantity, water quality, and disaster, the improvement of water quality and disaster prevention capacity is obvious in the three basins. Among them, the level of WPVI has increased by two levels in the Huang River Basin, while the level of WDVI has increased by two levels in the Hai River Basin. Second, through the analysis of the causes of the WRV, we found that the water resources situation of the Huang River Basin is better than the other two basins, and the causes of vulnerability are not significant. Furthermore, the critical vulnerability of the Huai River Basin is caused by water quality, while the critical vulnerability of the Hai River Basin is caused by disasters. So, the governance planning should be based on the reasons for the critical vulnerability of the three basins. Thirdly, from the analysis of the scenario prediction of WRV, if the three basins are developed according to scenario 1, the water resource situation will be greatly improved, and the WRV in the Huai and Hai River Basins is expected to reach moderate to low vulnerability (level 3) in 2030, and even reach mild vulnerability (level 2) in the Huang River Basin.

To summary up, this study has following contributions. First and foremost, this study for the first time used the index system method to evaluate the WRV in the Huang-Huai-Hai Basin in a combined study. Specifically, by establishing an evaluation index system, we have examined the WRV from the aspects of water shortage, water pollution, and natural hazards between 2000 and 2015. A number of empirical evidences regarding water resources vulnerability are revealed for this river basin system, which is of great strategic importance in China. Second, based on a reduced-dimension index system, we have trained a RF model that can simulate the evaluation process based on the full index system. This newly trained random forest model has a more concise structure but can retain the evaluation precision as the full model, which provides a more convenient tool for further study to evaluate water resources vulnerability in this region. Third, other than solely assessing the current condition for water resources vulnerability in this region, we also projected how the water resources vulnerability will be changing under three different climate change scenarios. These new findings indicated that the water resources vulnerability may deteriorate in this region under certain climate change conditions, which provide more evidence calling for the efforts to curb climate change. 

Influenced by climate conditions, geographical location, and other comprehensive factors, there are many problems in water resources in the Huang-Huai-Hai Basin, and the situation of water resources management is complex and severe. In the future, we will continue to pay attention to the development of WRV in the three river basins as more data come in over time. 

## Figures and Tables

**Table 1:** Table of the evaluation index system. WSVI: water shortage vulnerability, WPVI: water pollution vulnerability, WDVI: water-related natural disaster vulnerability, GDP: Gross Domestic Product, COD: Chemical Oxygen Demand.

		Evaluation Index System	Attribute
WSVI	Pressure	Water production modulus *A_1_*	positive
		Variation coefficient of annual precipitation *A_2_*	negative
	State	Proportion of groundwater supply *A_3_*	negative
		Change rate of annual precipitation *A_4_*	positive
	Impact	Utilization rate of surface water resources *A_5_*	negative
		Utilization rate of groundwater resources *A_6_*	negative
	Response	Per capita water consumption *A_7_*	negative
		Water consumption per mu *A_8_*	negative
WPVI	Pressure	Population density *B_1_*	negative
		Waste water discharge per 10,000 yuan GDP *B_2_*	negative
	State	Qualification rate of water quality in water function area *B_3_*	positive
		Qualification rate of water quality in river basin *B_4_*	positive
	Impact	Water consumption for ecosystem *B_5_*	positive
		Qualified decline rate of water quality *B_6_*	negative
	Response	COD emission per 10,000 people *B_7_*	negative
		Ammonia and nitrogen emission per 10,000 people *B_8_*	negative
WDVI	Pressure	Population carrying capacity per 10,000 m^3^ water *C_1_*	negative
		Reclamation index *C_2_*	negative
	State	Proportion of disaster area caused by drought and flood *C_3_*	negative
		Water yield coefficient *C_4_*	positive
	Impact	Proportion of effective irrigation area *C_5_*	positive
		Proportion of population under dike protection *C_6_*	positive
	Response	Control rate of soil erosion *C_7_*	positive
		Regulation capacity of water conservancy project *C_8_*	positive

**Table 2:** Threshold table of evaluation indexes of water resources vulnerability (WRV) in Huang-Huai-Hai Basin.

Interval Value of Vulnerability				Grade of Vulnerability
0 ≤ WVI < 0.133	0 ≤ WSVI < 0.049	0 ≤ WPVI < 0.046	0 ≤ WDVI < 0.039	No vulnerability (1^st^ level)
0.133 ≤ WVI < 0.247	0.049 ≤ WSVI < 0.096	0.046 ≤ WPVI < 0.077	0.039 ≤ WDVI < 0.074	Mild vulnerability (2^nd^ level)
0.247 ≤ WVI < 0.369	0.096 ≤ WSVI < 0.150	0.077 ≤ WPVI < 0.109	0.074 ≤ WDVI < 0.110	Moderate to low vulnerability (3^rd^ level)
0.369 ≤ WVI < 0.483	0.150 ≤ WSVI < 0.195	0.109 ≤ WPVI < 0.141	0.110 ≤ WDVI < 0.147	Moderate vulnerability (4^th^ level)
0.483 ≤ WVI < 0.601	0.195 ≤ WSVI < 0.240	0.141 ≤ WPVI < 0.178	0.147 ≤ WDVI < 0.183	Moderate to high vulnerability (5^th^ level)
0.601 ≤ WVI < 0.731	0.240 ≤ WSVI < 0.285	0.178 ≤ WPVI < 0.225	0.183 ≤ WDVI < 0.222	Highly vulnerability (6^th^ level)
0.731 ≤ WVI < 1	0.285 ≤ WSVI < 1	0.225 ≤ WPVI < 1	0.222 ≤ WDVI < 1	Extreme vulnerability (7^th^ level)

**Table 3:** Comparison accuracy of the two training models. MSE: Mean Square Error, NMSE: Normalized Mean Square Error.

Model	MSE	NMSE
RF	0.0008	0.2197
ANN	0.0016	0.6124

**Table 4:** Assessment of water resources vulnerability in the Huang-Huai-Hai Basin.

River Basin	Year	WVI	WVI (Level)	WSVI	WSVI (Level)	WPVI	WPVI (Level)	WDVI	WDVI (Level)
Huang River Basin	2000	0.4529	4	0.1537	4	0.1749	5	0.1269	4
	2001	0.4570	4	0.1509	4	0.1794	6	0.1251	4
	2002	0.4585	4	0.1579	4	0.1851	6	0.1228	4
	2003	0.4432	4	0.1379	3	0.1757	5	0.1228	4
	2004	0.4313	4	0.1476	3	0.1652	5	0.1197	4
	2005	0.3881	4	0.1313	3	0.1569	5	0.1107	4
	2006	0.4186	4	0.1492	3	0.1576	5	0.1252	4
	2007	0.3574	3	0.1096	3	0.1458	5	0.0970	3
	2008	0.3627	3	0.1126	3	0.1457	5	0.0996	3
	2009	0.3398	3	0.0962	2	0.1390	4	0.0934	3
	2010	0.3285	3	0.0966	3	0.1355	4	0.0950	3
	2011	0.3286	3	0.1030	3	0.1201	4	0.1131	4
	2012	0.3169	3	0.0944	2	0.1112	4	0.1061	3
	2013	0.3262	3	0.0969	3	0.1116	4	0.1098	3
	2014	0.3310	3	0.0987	3	0.1076	3	0.1173	4
	2015	0.3716	4	0.1218	3	0.1071	3	0.1336	4
Huai River Basin	2000	0.4825	4	0.1230	3	0.2078	6	0.1466	5
	2001	0.5205	5	0.1630	4	0.2054	6	0.1592	5
	2002	0.4638	4	0.1308	3	0.1987	6	0.1407	4
	2003	0.4544	4	0.1228	3	0.2043	6	0.1383	4
	2004	0.4447	4	0.1248	3	0.1930	6	0.1333	4
	2005	0.4346	4	0.1218	3	0.1871	6	0.1325	4
	2006	0.4246	4	0.1063	3	0.1765	5	0.1339	4
	2007	0.4036	4	0.1033	3	0.1779	5	0.1270	4
	2008	0.3917	4	0.1016	3	0.1644	5	0.1287	4
	2009	0.4318	4	0.1132	3	0.1682	5	0.1399	4
	2010	0.3865	4	0.1033	3	0.1577	5	0.1258	4
	2011	0.3936	4	0.1142	3	0.1563	5	0.1235	4
	2012	0.4085	4	0.1210	3	0.1571	5	0.1302	4
	2013	0.4191	4	0.1227	3	0.1566	5	0.1397	4
	2014	0.3978	4	0.1121	3	0.1470	5	0.1404	4
	2015	0.3852	4	0.1012	3	0.1491	5	0.1352	4
Hai River Basin	2000	0.5773	5	0.1755	4	0.1744	5	0.2227	7
	2001	0.5872	5	0.1883	4	0.1689	5	0.2219	7
	2002	0.5917	5	0.1914	4	0.1639	5	0.2221	7
	2003	0.5372	5	0.1722	4	0.1563	5	0.1989	6
	2004	0.4856	5	0.1509	4	0.1554	5	0.1832	6
	2005	0.5041	5	0.1680	4	0.1550	5	0.1849	6
	2006	0.5187	5	0.1803	4	0.1533	5	0.1916	6
	2007	0.5181	5	0.1712	4	0.1487	5	0.1965	6
	2008	0.5022	5	0.1578	4	0.1439	5	0.1861	6
	2009	0.5093	5	0.1621	4	0.1405	4	0.1977	6
	2010	0.4589	4	0.1483	3	0.1381	4	0.1678	5
	2011	0.4639	4	0.1532	4	0.1390	4	0.1646	5
	2012	0.4476	4	0.1496	3	0.1380	4	0.1582	5
	2013	0.4360	4	0.1499	3	0.1361	4	0.1469	5
	2014	0.4531	4	0.1608	4	0.1394	4	0.1528	5
	2015	0.4393	4	0.1603	4	0.1257	4	0.1685	5

**Table 5:** Scenario prediction of WRV in Huang-Huai-Hai Basin.

River Basin	Scene	Year	WVI	WVI (Level)	WSVI	WSVI (Level)	WPVI	WPVI (Level)	WDVI	WDVI (Level)
Huang River Basin	Scene1	2020	0.2731	3	0.1301	3	0.0781	3	0.0933	3
		2030	0.2312	2	0.1307	3	0.0507	2	0.0721	2
	Scene2	2020	0.3348	3	0.1362	3	0.1085	3	0.1062	3
		2030	0.2986	3	0.1399	3	0.0896	3	0.0979	3
	Scene3	2020	0.3572	3	0.1620	4	0.1124	4	0.1180	4
		2030	0.3594	3	0.1640	4	0.1087	3	0.1173	4
Huai River Basin	Scene1	2020	0.3463	3	0.1198	3	0.1078	3	0.1221	4
		2030	0.3082	3	0.1201	3	0.0853	3	0.0828	3
	Scene2	2020	0.3827	4	0.1113	3	0.1355	4	0.1368	4
		2030	0.3231	3	0.1036	3	0.0881	3	0.0866	3
	Scene3	2020	0.4040	4	0.1260	3	0.1528	5	0.1402	4
		2030	0.3739	4	0.1264	3	0.1231	4	0.1330	4
Hai River Basin	Scene1	2020	0.4033	4	0.1587	4	0.1048	3	0.1508	5
		2030	0.3386	3	0.1617	4	0.0900	3	0.0993	3
	Scene2	2020	0.4402	4	0.1641	4	0.1253	4	0.1614	5
		2030	0.4064	4	0.1647	4	0.1206	4	0.1178	4
	Scene3	2020	0.4766	4	0.1673	4	0.1400	4	0.1719	5
		2030	0.4251	4	0.1669	4	0.1286	4	0.1369	4

## Appendix A. Scenario Prediction Data of Indicators in the Huang-Huai-Hai River Basin

**Table A1:** Scenario prediction data of indicators in the Huang-Huai-Hai river basin.

River Basin	Scene	Year	*A_1_*	*A_6_*	*A_7_*	*A_8_*	*B_2_*	*B_3_*	*B_4_*	*B_5_*	*C_3_*	*C_6_*	*C_7_*	*C_8_*
Huang River Basin	Scene1	2020	8.84	0.30	459.26	398.00	2.60	0.80	0.69	0.05	0.10	0.73	0.83	0.97
		2030	8.43	0.30	430.71	361.00	2.00	0.95	0.76	0.06	0.10	0.68	0.95	1.06
	Scene2	2020	8.29	0.34	459.26	419.00	2.99	0.56	0.63	0.04	0.11	0.68	0.67	0.88
		2030	8.16	0.36	430.71	373.00	2.99	0.67	0.69	0.06	0.11	0.66	0.76	0.97
	Scene3	2020	8.54	0.38	486.96	465.00	5.00	0.48	0.56	0.03	0.13	0.48	0.50	0.79
		2030	7.56	0.40	456.69	465.00	5.00	0.48	0.62	0.05	0.13	0.48	0.57	0.87
Huai River Basin	Scene1	2020	32.64	0.40	275.38	256.50	5.00	0.80	0.51	0.02	0.07	0.58	0.74	0.31
		2030	31.02	0.34	278.96	234.00	3.37	0.95	0.61	0.03	0.04	0.74	1.00	0.34
	Scene2	2020	32.04	0.42	327.06	286.00	7.36	0.72	0.46	0.02	0.08	0.55	0.67	0.28
		2030	29.46	0.38	331.30	256.00	5.00	0.86	0.56	0.03	0.04	0.67	0.90	0.31
	Scene3	2020	31.19	0.47	339.94	316.00	9.70	0.64	0.41	0.02	0.09	0.52	0.60	0.26
		2030	28.75	0.42	344.35	316.00	10.80	0.76	0.50	0.03	0.05	0.59	0.70	0.28
Hai River Basin	Scene1	2020	10.13	0.97	353.04	249.50	2.32	0.80	0.40	0.06	0.19	0.86	0.20	0.26
		2030	10.45	0.90	337.09	232.00	2.00	0.95	0.44	0.08	0.15	0.96	0.80	0.33
	Scene2	2020	9.30	1.00	357.20	299.50	2.87	0.48	0.36	0.05	0.22	0.78	0.18	0.23
		2030	9.69	1.00	341.06	276.00	2.87	0.57	0.40	0.08	0.17	0.87	0.72	0.30
	Scene3	2020	9.76	1.13	367.60	323.00	5.00	0.29	0.33	0.04	0.28	0.70	0.16	0.21
		2030	9.61	1.10	350.99	323.00	5.00	0.48	0.36	0.06	0.22	0.77	0.56	0.27

The specific calculation method of scenario forecast data of each indicator is as follows.

### Appendix A1. Water Production Modulus A_1_

This indicator is mainly influenced by natural factors such as climate change. The water production modulus is equal to the total amount of basin water resources divided by the area of the basin.

The total amount of basin water resources is equal to the total annual precipitation multiplied by the coefficient of water production, and the total annual precipitation data could be obtained by the existing research in three different scenarios, which could be found in [36] (p. 145–146). However, the figures of the water yield coefficient change due to the underlying surface change, in which the three basins show different degrees of change. According to the research, the annual water coefficient of the Huai river basin decreased by 0.02 per 10 years, while that of the Huang and the Hai river basins decreased by 0.06 per 10 years.

In the opinion of these methods, the water yield modulus figures of the three basins could be predicted in 2020 and 2030 under three scenarios.

### Appendix A2. Utilization Rate of Groundwater Resources A_6_

This indicator is mainly influenced by both climate and social-economic development.

First of all, the indicator data of three river basins show no obvious trends in the period 2000–2015. The data will be processed in the following manner. Firstly, the average value of this index in three basins over 16 years was calculated, and the minimum value was found during 2000–2015. Then, according to the water resources planning goals of 2020 and 2030 of the three basins, and the corresponding realization ratio, the scenario prediction is carried out.

For example, the 16-year average of the Huang River Basin is 0.3632 and the minimum value is 0.3039. So, in 2020 and 2030, scenario 1 is projected by the minimum of 0.3039, and scenario 2 is projected by the average. However, scenario 3 is calculated through multiplying scenario 2 by the coefficient, which assumes that it is calculated on the figure over 10% in scenario 2. The same calculation was used for the remaining two basins.

### Appendix A3. Per Capita Water Consumption A_7_

This indicator is mainly influenced by social-economic activities. According to the definition of the indicator, it is easy to know that the indicator value is equal that the total water consumption of the basin divided by the total population of the basin.

For the total water consumption of the basin, the data in 2020 and 2030 under scenario 1 can be predicted according to the target of three red lines in the SWRMS [35]. In addition, the control objectives of the total water consumption of the three basins should be referred to. The total population of the basin uses the existing research results [36]. So, the index value could under scenario 1 be predicted under in this way. In addition, scenario 2 and scenario 3 are calculated according to the ratio of the predicted total basin water consumption to the predicted population.

### Appendix A4. Water Consumption per Mu A_8_

This indicator is mainly influenced by natural factors such as climate change, especially the current year’s rainfall.

The predicted value of this indicator under scenario 1 is mainly the average water consumption per mu in the normal year (normal precipitation year) adopted, the indicator data is assumed for moderately dry years under scenario 2, and the indicator data is assumed for dry years under scenario 3. These scenario data can be found on page 125 of the existing literature [36].

### Appendix A5. Waste Water Discharge per 10,000 Yuan GDP B_2_

This indicator is mainly influenced by human production activities, sewage treatment, and so on.

The data of this index in three river basins have a trend of decreasing year by year during 2000–2015. So, our three scenarios are predicted in different ways, as follows. Scenario 1 predictive analysis is implemented using the strictest standards of the three red lines in the strictest water resources system in 2020 and 2030 [35]. Scenario 2 is calculated at the normal rate of decline, while scenario 3 data is calculated at the rate of deceleration, which is 20% slower than that of scenario 2.

### Appendix A6. Qualification Rate of Water Quality in Water Function Area B_3_

This indicator is mainly influenced by socio-economic development activities.

First, the trends in the mean and indicators of the three basins were analyzed during 2000–2015. The average of this index for 16 years in the three basins of the Huang-Huai-Hai river basins are 0.3838, 0.4288, and 0.2859. According to the trend analysis of the index value, the index values of the Huang River Basin and the Huai River Basin have always had an upward trend. However, there is no obvious improvement trend of the index of the Hai River Basin. Based on this analysis, we forecast three scenarios in 2020 and 2030 for three basins. Second, according to the analysis of the three red-line goals of the SWRMS, the three basins are considered to meet the target requirements of the three red lines by 2020 and 2030 under scenario 1, which is the compliance rate target of 80% and 95%. Scenario 2 and scenario 3 of the three basins are determined on the basis of the scenario 1 prediction data, which is following the proportion of each realization.

### Appendix A7. Qualification Rate of Water Quality in River Basin B_4_

This indicator is the proportion of the basins’ water spread among 1–3 types and mainly influenced by socio-economic activities.

First, the trend of this indicator has increased year by year in the three basins between 2000 and 2015. In addition, this indicator not has a specific target value in the SWRMS. So, the forecast of the indicator is mainly based on the development trend. Specifically, the value of scenario 2 should be determined first. In scenario 2, the values for 2020 and 2030 are measured at the annual growth rates of the three basins. Based on scenario 2, scenario 1 increases by 10%; scenario 3 decreases by 10%.

### Appendix A8. Water Consumption for Ecosystem B_5_

This indicator is mainly influenced by the socio-economic activities.

First, the trend of this indicator has increased year by year in the three basins during 2000–2015. So, the scenario prediction of this indicator is mainly based on the development trend. The value of scenario 2 should be determined first. According to the growth rate of this indicator, the forecast values of three basins under scenario 2 in 2020 and 2030 can be calculated respectively. Based on scenario 2, scenario 1’s projection increases by 10%; scenario 3’s projection decreases by 10%.

### Appendix A9. Proportion of Disaster Area Caused by Drought and Flood C_3_

This indicator is mainly influenced by both natural factors and human social activities.

Except for individual years, the trend of this indicator has decreased year by year in the three basins between 2000 and 2015. It could be explained that the disaster prevention capacity of water conservancy projects is gradually increasing. It is more scientific to use the average of nearly five years to predict scenarios for future years. It could calculate the average for nearly five years as scenario 2 for 2020, while scenario 1 and scenario 3 increase and decrease by 10% respectively on the basis of scenario 2.

### Appendix A10. Proportion of Population under Dike Protection C_6_

This indicator is mainly influenced by human economic and social activities, which are mainly related to the construction of a flood control project in the basin.

The data trend of the three river basins from 2000 to 2015 has been analyzed. It can be found that the change trend of this data in the Huang River Basin is not obvious, while the Huai River Basin and the Hai River Basin data basically have a gradual upward trend.

Based on the trend analysis of the previous data, three river basins could be predicted. Firstly, the data of scenario 2 of the Huai River and Hai River Basin in 2020 could be predicted using the average of nearly 5 years. Scenarios 1 and 3 increase or decrease by 10%. The 2030 scenario 1 data for both basins use the highest values in the 16-year historical data, which is because the authors argue that conservation works for the population must be increasing with watershed governance. Scenario 2 of the Huang River Basin uses a 16-year average, while scenario 1 and scenario 3 increase or decrease by 10% on the basis of scenario 2.

### Appendix A11. Control Rate of Soil Erosion C_7_

This indicator is mainly influenced by human socio-economic activities, which are related to basin management measures.

First, the target values of indicator for the three basins could be found in the 2020 and 2030 planning reports. In scenario 1, the predicted values of indicators are mainly predicted according to the target values of the three basins. Scenario 2 and scenario 3 are speculated according to the realization ratio of scenario 1. However, the realization proportion of the three basins is different. Specifically, because the current value of this indicator in the Huai River Basin is not far from the target value, scenario 2 and scenario 3 are calculated as 0.9 and 0.7, respectively. However, the current value of this indicator in the Hai River Basin and Huang River Basin are far from the planned target values, so scenarios 2 and 3 are calculated according to 0.8 and 0.6 of scenario 1, respectively.

### Appendix A12. Regulation Capacity of Water Conservancy Project C_8_

The formula for this indicator is the year-end water storage of the reservoir divided by the total water supply. This indicator is mainly influenced by human socio-economic activities, which are related to the construction and management of reservoirs.

No obvious trend of change in data analysis of the three basins from 2000 to 2015 has been found, but the average of the last five years was larger than the average of the previous five years. These data have a large correlation with the reservoir capacity, so it has a large correlation with the recent data, and it has little correlation with the more distant data.

Based on this analysis, the forecast data of this indicator under scenario 2 are the average values of three basins in recent five years in 2020. Scenario 1 and scenario 3 increased and decreased by 10% respectively on the basis of scenario 2. In addition, the indicator data of scenario 2 in 2030 is the average of the past five years multiplied by the average 10-year growth rate in the historical data. Scenario 1 and scenario 3 increased and decreased by 10% respectively on the basis of scenario 2.
